# Prognostic Significance of Serum Inflammatory Response Markers in Newly Diagnosed Non-Small Cell Lung Cancer before Chemoirradiation

**DOI:** 10.1155/2015/485732

**Published:** 2015-08-03

**Authors:** Maria Tolia, Nikolaos Tsoukalas, George Kyrgias, Eftychia Mosa, Apostolos Maras, Ioannis Kokakis, Zoi Liakouli, John R. Kouvaris, Konstantinos Liaskonis, Nikolaos Charalampakis, Kyriaki Pistevou-Gombaki, Nikolaos Kelekis, Vassilis Kouloulias

**Affiliations:** ^1^Radiation Therapy Oncology Unit, Second Department of Radiology, University Hospital of Athens “ATTIKON”, Rimini 1, Haidari, 124 64 Athens, Greece; ^2^Oncology Clinic, 401 General Military Hospital, Mesogeion 138 & Katehaki, 115 25 Athens, Greece; ^3^Department of Radiotherapy, Faculty of Medicine, School of Health Sciences, University of Thessaly, Biopolis, 41110 Larissa, Greece; ^4^Pammakaristos General Hospital, Iakovaton 43, 11144 Athens, Greece; ^5^Radiation Therapy Oncology Department, First Department of Radiology, Aretaieion Hospital, University of Athens Medical School, Vassilissis Sofias 76, 115 28 Athens, Greece; ^6^Biochemistry Department, 401 General Military Hospital, Mesogeion 138 & Katehaki, 115 25 Athens, Greece; ^7^Oncology Clinic, University Hospital of Athens “ATTIKON”, Rimini 1, Haidari, 124 64 Athens, Greece; ^8^Radiation Oncology Clinic, University Hospital of Thessaloniki AHEPA, 1 Street Kyriakidi, 54636 Thessaloniki, Greece

## Abstract

*Purpose*. To identify whether the serum's baseline C-reactive protein (CRP) and albumin (Alb) levels related to clinicopathological parameters and overall survival (OS) in non-small cell lung cancer (NSCLC). *Methods*. In total, 100 consecutive patients (mean age = 68.38 ± 10.85 years) that underwent chemoradiotherapy were studied. Measurements of CRP and Alb were performed before any treatment. *Results*. Serum CRP levels were significantly associated with histological grade (*P* < 0.001), TNM stage (*P* < 0.001), PS (*P* = 0.009), and Alb (*P* < 0.001). Additionally CRP and Alb levels were found significantly associated with overall survival in univariate analysis (log-rank test, *P* < 0.001 and *P* = 0.002, resp.) and CRP remained significant after controlling for age, alcohol, performance status, and TNM stage, whereas albumin showed a borderline effect on the hazard rate (*P* = 0.052). *Conclusions*. CRP and Alb are both promising biomarkers in identification of NSCLC patients with poor prognosis and form a possible target for intensifying their therapies.

## 1. Introduction

Systemic inflammation increases cell proliferation because it promotes neoplastic risk [1]. Genetic events, as an intrinsic pathway, and inflammatory condition as an extrinsic pathway can predispose to neoplasia [2]. Cancer-related chronic inflammation affects DNA damage, continuous replication, sustained angiogenesis, apoptosis evasion, self-sufficiency in growth signaling, insensitivity to antigrowth signaling, and tissue invasion/metastasis [3].

Many different tumor-associated factors have been described and investigated for lung cancer. The identification of markers whose altered expression is correlated with OS differences might enclose the knowledge to distinguish those which could serve as indicators of the tumor's biological behavior. CRP and Alb are acute phase proteins and their concentrations are related to the presence of an inflammation or neoplasm. CRP offers a reliable clinical information on the active inflammatory status due to its rapid variability [4, 5]. Alb serves as a splanchnic function indicator protein that in case of inflammation or hypothrepsia its synthesis is suppressed. Because of a tumor presence, a systemic inflammatory reaction is created and cytokines that induce acceleration of catabolism are released. Particularly interleukin-6 (ΙL-6) and interleukin-Ib (IL-Ib) decrease Kupffer cells' Alb production [4].

In this study, we employed nephelometric and photometric methods to evaluate serum CRP and Alb levels in NSCLC patients before chemoirradiation. Furthermore, we analyzed the correlation between CRP and Alb and variable clinicopathological features and patient prognosis.

## 2. Materials and Methods

All participating patients signed the informed consent. The inclusion criteria in the study were (a) PS according to the Zubrod Scale: 0–2, (b) newly diagnosed NSCLC (according to the TNM system), (c) no prior history of chemotherapy or RT, and (d) absence of acute inflammation signs.

A three-dimensional conformal radiotherapy (3DCRT) technique was used. The target volumes were defined according to ICRU Reports 50, 62 [6]. The organs at risk (OARs) and the dose constraints were determined by ICRU Report 62 and QUANTEC [6, 7]. A biologically equivalent dose equal to 60 Gy was delivered, in daily photon radiation fractions from Monday to Friday. Chemotherapy was administered according to the current NCCN (National Comprehensive Cancer Network) guidelines criteria [8, 9].

A peripheral blood sample was collected and centrifuged before starting any therapy. Serum CRP levels were measured using nephelometric method (Beckman Coulter, Image Immunochemistry System, USA), while serum Alb levels were determined using photometric method. Continuous data were presented as mean ± standard deviation, whereas categorical data were presented as absolute and relative frequency. Kolmogorov-Smirnov test evaluated the assumption of normality. To assess the differences of study parameters according to the levels of Alb and CRP, standard statistical procedures were used, as appropriate (Student's *t*-test for continuous data, Chi-square test for categorical data, and Fisher's Exact test for categorical data with limited number of frequencies). Survival curves were generated by Kaplan-Meier analysis and tested for significance using the Mantel-Cox log-rank test. Further on, Cox proportional regression analysis was used to identify potential independent prognostic marker. The SPSS statistical package (Version 20.0, IBM Corp.) was used to analyze the data. Significance level was set at *P* = 0.05 and Bonferroni-Holm correction was applied to compare differences between groups.

## 3. Results

**Figure 1:**
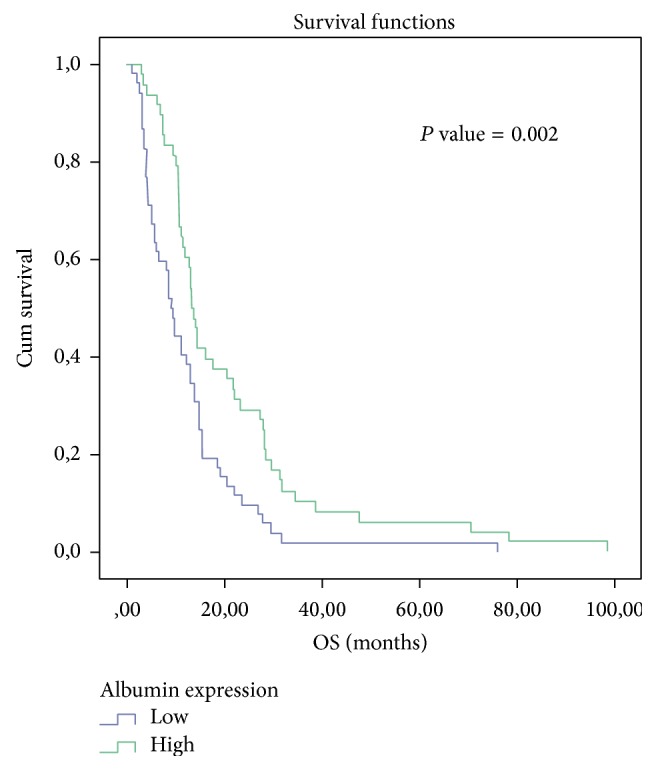
Kaplan-Meier survival analysis of Alb.

The study sample consisted of 100 participants. The mean age of the total sample was estimated at 68.38 ± 10.85 years, ranging between 36 and 92 years. The cut point for albumin (median: 3.5, IQR: 0.775) and CRP (median: 23.1, IRQ: 48.93), range 25%–75% levels was according to the median value (3.5 and 23.1, resp.).  Table 1 presents the distribution of the study variables according to the levels of Alb and CRP. There was no evidence for a possible association between Alb and presence of NEC (*P* = 0.108) and FIB (*P* = 0.149), smoking status (*P* = 0.439), ETOH consumption (*P* = 0.275), presence of INF (*P* = 0.894) and LVI (*P* = 0.894), performance status (*P* = 0.036), and TNM stage (*P* = 0.012) after *P* value adjustment with Bonferroni-Holm's correction. On the other hand, we found evidence that albumin levels are associated with patients age (*P* = 0.002). We also find evidence that CRP is strongly associated with histological grade (*P* < 0.001), TNM stage (*P* < 0.001), and OS (*P* < 0.001). The comparison of survival with Kaplan-Meier survival analysis between low Alb levels (blue line, *n* = 52) and high Alb levels (green line, *n* = 48) showed a statistically significant better prognosis for high levels of albumin (*P* = 0.002) (Figure 1). The median time for patients with lower albumin levels was 9.167 ± 0.821 (95% CI: 7.557–10.776) versus 13.267 ± 0.759 (95% CI: 11.779–14.754) for the patients with higher levels of albumin.  Table 1 shows the distribution of 100 participants according to the Alb and CRP levels.  Table 2 shows the results from the univariate Cox regression analysis examining the relationship between overall survival and Alb.

**Figure 2:**
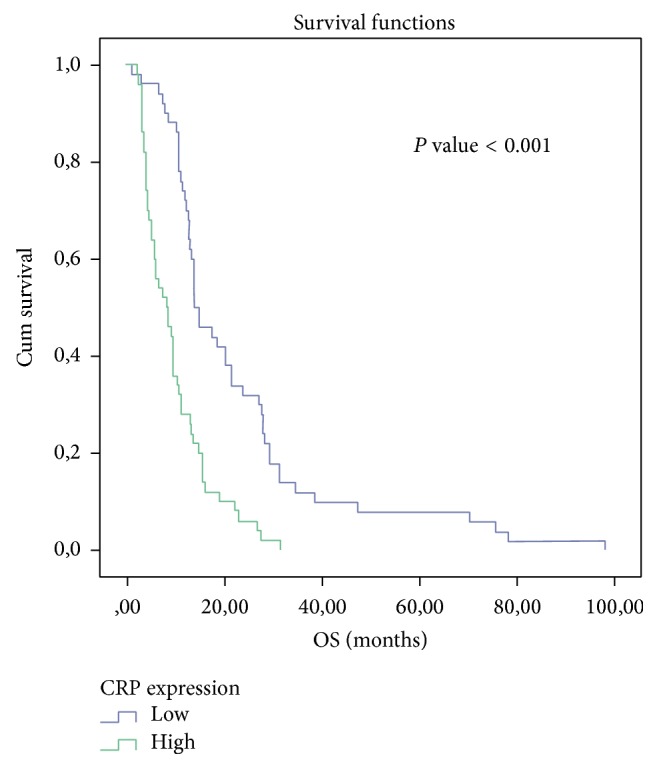
Kaplan-Meier survival analysis of CRP.

The comparison of survival with Kaplan-Meier survival analysis between low CRP levels (blue line, *n* = 50) and high CRP levels (green line, *n* = 50) demonstrated a statistically significant better prognosis for low CRP levels (*P* < 0.001) (Figure 2). The median time for patients with lower CRP levels was 14.167 ± 2.220 (95% CI: 9.816–18.517) versus 8.133 ± 1.827 (95% CI: 4.553–11.714) for the patients with higher CRP levels.  Table 3 presents the univariate Cox regression analysis examining the relationship between overall survival and CRP.

Table 4 shows a univariate analysis for all parameters. A multiple Cox regression analysis examined the relationship between overall survival and Alb, after adjustment for demographic, clinical, and histological parameters in the total sample of 100 participants. According to the findings, Alb seems to have a borderline effect on the hazard rate (*P* = 0.052). Other parameters that were found to be highly associated with the hazard rate were heavy drinkers with a hazard ratio of 1.767 versus nonsocial drinkers (*P* = 0.017), TNM stage with a hazard ratio of 2.506 (*P* = 0.001), and performance status with a hazard ratio 2.602 (*P* = 0.001).

Table 5 demonstrates a multiple Cox regression analysis examining the relationship between overall survival and CRP, after adjustment for demographic, clinical, and histological parameters in the total sample of 100 participants. According to the findings, CRP seems to have a significant effect on the hazard rate (*P* = 0.002). Other parameters that were found to be highly correlated with the hazard rate were heavy drinkers with a hazard ratio of 1.690 versus social drinkers (*P* = 0.024), TNM stage with a hazard ratio of 2.238 (*P* = 0.004), and performance status with a hazard ratio of 2.407 (*P* = 0.002).

## 4. Discussion

In the present study, we found a correlation between CRP and Alb with survival in NSCLC. CRP and Alb are easily obtainable biomarkers associated with the lung parenchyma lesion, caused by the tumor presence. The lower CRP baseline and the elevated Alb baseline values were correlated with better outcome in terms of OS (log-rank test *P* < 0.001 and *P* = 0.002, resp.). The combination of the increased CRP values and hypoalbuminemia may be due to one of the following: (a) patients' malnutrition (hypothrepsia) or (b) reactive response (tissue stress) due to the existence of cancer cells that activate the production of acute phase proteins [10]. McMillan et al. demonstrated that CRP may be a significant independent predictor of OS in advanced cancer patients (*P* = 0.0002) [11]. Siemes et al. [12] found that baseline CRP levels seemed to be a biomarker of chronic inflammation preceding lung cancer, even after subtracting a 5-year latent period (HR = 2.8; 95% CI = 1.6–4.9). Allin and Nordestgaard [13] demonstrated that individuals with CRP levels in the highest versus the lowest quintile had a 2-fold increased risk of lung cancer. Among individuals diagnosed with cancer, patients with a high baseline CRP (>3 mg/L) had an 80% greater risk of early death versus those with low CRP levels (<1 mg/L). Roxburgh and McMillan [14] showed that, in primary operable cancer, preoperative estimation of the systemic inflammatory response such as elevated CRP, hypoalbuminemia or increased white cell, neutrophil, and platelet counts predicted cancer OS regardless of the tumor stage. O'Dowd et al. [15] indicated that preoperative CRP more than 34 mg/L (HR = 1.65, 95% CI = 1.12–3.87, *P* = 0.045) retained independent significance of poor outcome in ninety-six lung cancer patients. CRP levels >10 mg/L had a median OS of 26.2 months versus 75.9 months of those patients with a CRP < or = 10 mg/L (*P* < 0.05). In our previous study [16] we found that CRP, Ferritin, and Alb were correlated with the acute complication of lung parenchyma radiation induced toxicity. CRP and Ferritin were elevated in the immediate postradiotherapy interval (after 2 months) compared to the preradiotherapy values (*P* < 0.001). The Alb levels were found to be lower (*P* < 0.001). Pine et al. [17] showed that the 10-year standardized absolute risk of lung cancer was the highest among current smokers with high IL-8 and CRP levels (absolute risk = 8.01%, 95% CI = 5.77% to 11.05%). Xu et al. [18] found that higher levels of CRP were associated with increasing lung cancer risk (OR = 2.11, 95% CI = 1.66–2.91, *P* < 0.01), suggesting that CRP could be used as surrogate biomarker of angiogenesis and prognosis in lung cancer. We observed a correlation between CRP and PS (*P* = 0.009), LVSI (*P* = 0.009), TNM stage (*P* < 0.001), and OS (*P* < 0.001). Accordingly Tulek et al. [19] also found that CRP levels were significantly elevated (*P* = 0.001) in NSCLC patients with poor PS. Higgins et al. [20] associated LVSI with an increased risk of harboring regional lymphonodal involvement (*P* < 0.001). LVSI was also an adverse prognostic factor for the development of distant metastases (*P* = 0.006) and long-term survival (*P* = 0.003) in adenocarcinomas. The analysis of our data indicated significantly worse OS for lung cancer patients with hypoalbuminemia from survival analysis*:* log-rank test (*P* = 0.002), univariate Cox regression (*P* = 0.003), and multiple Cox regression (borderline *P* = 0.052). In multivariate analysis lower levels of Alb were linked with stage of disease (*P* = 0.012), the elderly (*P* = 0.002), and performance status (*P* = 0.036). Jin et al. [21] had already identified preoperative and postoperative hypoalbuminemia (<3.5 g/dL) as independent negative prognostic factors for recurrence (*P* = 0.008 and *P* = 0.001, resp.).

## 5. Conclusion

The present study provides evidence that higher pretreatment CRP and lower Alb serum levels are potential prognostic factors of OS. Our data could be useful to improve risk stratification and to develop better tailored treatment strategies in NSCLC patients.

## Figures and Tables

**Table 1:** Distribution of 100 participants according to the expression of Alb and CRP.

Variables	Alb expression		*P* value	CRP expression		*P* value
	Low expression	High expression		Low expression	High expression	
	(*N* = 52)	(*N* = 48)		(*N* = 50)	(*N* = 50)	
Age, years	71.58 ± 10.11	64.92 ± 10.66	**0.002** ^†^	66.34 ± 9.52	70.42 ± 11.77	0.060^†^
PS			0.036^††^			0.009^†††^
0-1	38 (73.1)	43 (89.6)		46 (92.0)	35 (70.0)	
2–4	14 (26.9)	5 (10.4)		4 (8.0)	15 (30.0)	
Smoking			0.439^†††^			0.99^†††^
No	5 (9.6)	2 (4.2)		3 (6.0)	4 (8.0)	
Smokers	47 (90.4)	46 (95.8)		47 (94.0)	46 (92.0)	
ETOH			0.275^††^			0.99^††^
Social	24 (46.2)	17 (35.4)		20 (40.0)	21 (42.0)	
Heavy	28 (53.8)	31 (64.6)		30 (60.0)	29 (58.0)	
INF			0.894^††^			0.99^††^
No	47 (90.4)	43 (89.6)		45 (90.0)	45 (90.0)	
Yes	5 (9.6)	5 (10.4)		5 (10.0)	5 (10.0)	
LVI			0.894^††^			0.009^†††^
No	47 (90.4)	43 (89.6)		46 (92.0)	35 (70.0)	
Yes	5 (9.6)	5 (10.4)		4 (8.0)	15 (30.0)	
NEC			0.108^††^			0.68^††^
No	42 (80.8)	32 (66.7)		33 (66.0)	41 (82.0)	
Yes	10 (19.2)	5 (10.4)		17 (34.0)	9 (18.0)	
FIB			0.149^†††^			0.059^†††^
No	42 (80.8)	32 (66.7)		43 (86.0)	49 (98.0)	
Yes	10 (19.2)	5 (10.4)		7 (14.0)	1 (2.0)	
TNM stage			0.012^††^			<0.001^††^
I and II	8 (15.4)	18 (37.5)		21 (42.0)	5 (10.0)	
III and IV	44 (84.6)	30 (62.5)		29 (58.0)	45 (90.0)	
Histological grade			0.005^††^			<0.001^††^
I and II	20 (38.5)	32 (66.7)		39 (78.0)	13 (26.0)	
III and IV	32 (64.5)	16 (33.3)		11 (22.0)	37 (74.0)	
OS	12.09 ± 11.83	20.90 ± 19.04	0.007^†^	22.92 ± 9.73	19.85 ± 7.07	<0.001^†^

PS = performance status, ETH = alcohol, INF = inflammation, LVI = lymphovascular invasion, NEC = necrosis, FIB = fibrosis, and OS = overall survival. Data are presented as *N* (%) or mean ± standard deviation. Bonferroni-Holm correction was applied to compare differences between groups. ^†^ *P* value derived from Student's *t*-test, ^††^ *P* value derived from Chi-square test, and ^†††^ *P* value derived from Fisher's Exact test.

**Table 2:** Univariate Cox regression analysis examining the relationship between overall survival and Alb.

Variable	B	SE⁡(*B*)	*P* value	Exp⁡(*B*)
Alb (high/low expression)	−0.621	0.207	**0.003**	0.537

**Table 3:** Univariate Cox regression analysis examining the relationship between overall survival and CRP.

Variable	*B*	SE⁡(*B*)	*P* value	Exp⁡(*B*)
CRP (high/low expression)	1.055	0.218	**<0.001**	2.873

**Table 4:** Multiple Cox regression analysis examining the relationship between overall survival and Alb, after adjustment for demographic, clinical, and histological parameters in the total sample of 100 participants.

Variables	*B*	SE⁡(*B*)	*P* value	Exp⁡(*B*)
Alb (high/low expression)	−0.436	0.224	**0.052**	0.647
Age > 65 (yes/no)	0.386	0.228	0.091	1.471
ETH (heavy/social)	0.569	0.238	**0.017**	1.767
PS (0-1/2–4)	0.956	0.289	**0.001**	2.602
TNM stage (III and IV/I and II)	0.919	0.275	**0.001**	2.506

ETH = alcohol; PS = performance status.

**Table 5:** Multiple Cox regression analysis examining the relationship between overall survival and CRP, after adjustment for demographic, clinical, and histological parameters in the total sample of 100 participants.

Variables	B	SE⁡(*B*)	*P* value	Exp⁡(*B*)
CRP (high/low expression)	0.722	0.233	**0.002**	2.059
Age > 65 (yes/no)	0.354	0.230	0.123	1.425
ETH (heavy/social)	0.525	0.232	**0.024**	1.073
PS (0-1/2–4)	0.878	0.288	**0.002**	2.407
TNM stage (III and IV/I and II)	0.806	0.282	**0.004**	2.238

ETH = alcohol; PS = performance status.
